# Time to first and recurrent missed video directly observed therapy sessions and associated factors among people with Tuberculosis in Kampala, Uganda

**DOI:** 10.3389/fdgth.2026.1815509

**Published:** 2026-09-01

**Authors:** Kelvin Bwambale, Drake Amutuheire, Damalie Nakkonde, Patrick Kaggwa, Esther Buregyeya, Sarah Zalwango, Sarah Nabukeera, John B. Isunju, Juliet N. Sekandi

**Affiliations:** 1 School of Public Health, Makerere University, Kampala, Uganda; 2 Department of Epidemiology and Biostatistics, College of Public Health, University of Georgia, Athens, GA, United States; 3 Global Health Institute, College of Public Health, University of Georgia, Athens, GA, United States; 4 Kampala Capital City Authority, Department of Public Health Service and Environment, Kampala, Uganda

**Keywords:** survival analysis, treatment adherence monitoring, Tuberculosis, Uganda, Video Directly Observed Therapy

## Abstract

**Background:**

Video Directly Observed Therapy (VDOT) provides a promising alternative to in-person directly observed therapy (DOT) for Tuberculosis (TB) treatment adherence monitoring. However, sustained patient engagement and missed video submissions remain a challenge. This study examined the time to first and recurrent missed VDOT submissions and the associated factors.

**Methods:**

We conducted a retrospective cohort analysis study of 71 people with TB assigned to the VDOT arm of the DOT Selfie randomized trial in Kampala, Uganda. Primary outcomes were time to first and recurrent missed VDOT sessions, analyzed using the Kaplan–Meier estimator. Associated factors were identified with Cox regression and the Prentice-Williams-Peterson Gap Time model. Median times with corresponding first and third quartiles (Q1, Q3) and adjusted hazard ratios (aHRs) with their 95% Confidence Intervals were reported. Analyses were conducted in Stata 14·2.

**Results:**

Of the 71 participants, 36 (51%) were female, the median age was 29 years (24, 42), and 23 (32%) were living with HIV. The median time to the first missed VDOT session was 9 days (2, 39), and to recurrent missed sessions was 2 days (1, 8). Earlier first missed sessions were associated with HIV co-infection (aHR=1·99; 95% CI: 1·09, 3·62), being married (aHR = 1·88; 95% CI: 1·03, 3·42), and reporting alcohol use (aHR=4·11, 95% CI: 1·89, 8·97). Factors associated with earlier recurrences of missed sessions included being currently married (aHR = 1·34; 95% CI: 1·03, 1·75) compared to the never married. A larger household size was slightly protective against earlier recurrent missed sessions (aHR = 0·96; 95% CI: 0·92, 0·99).

**Conclusion:**

This study highlights the factors that are associated with earlier missed VDOT sessions in the Ugandan setting. These factors may help identify groups that could benefit from targeted adherence support in future VDOT implementation efforts. We recommend more research to understand the interplay of marital status with VDOT use.

## Introduction

Tuberculosis (TB) is the leading infectious cause of death worldwide, with an estimated 10·8 million new cases and 1·25 million deaths, according to the 2024 Global Tuberculosis Report (1). The World Health Organization (WHO) has set a target to eliminate TB by 2035 (2). However, non-adherence to TB treatment is a major barrier to achieving this target (1). To improve adherence monitoring, Uganda has traditionally relied on DOT, where healthcare workers or community supporters physically supervise patients as they take their medication. While effective, DOT presents significant logistical, financial, and feasibility challenges. These challenges include the need for a large workforce, long travel distances for both patients and providers, scheduling conflicts, and stigma associated with in-person supervision (3–5). These barriers contribute to persistent non-adherence despite DOT implementation. VDOT has emerged as a more patient-centered and scalable alternative, enabling patients to record and submit videos of their medication intake for remote monitoring, reducing the burden of in-person supervision while maintaining adherence oversight (6, 7).

However, the effectiveness of VDOT may be limited by missed video submissions to enable the health providers to monitor adherence. For example, a pilot cohort study conducted in Uganda that involved 50 people with TB over six months recorded over 17% of expected doses as missed video submissions (8). While existing studies have primarily assessed the acceptability and overall effectiveness of VDOT as compared to DOT, they have not examined the patterns and timing of missed video submissions. Understanding these patterns is essential because frequent or prolonged gaps in video submissions may serve as a proxy indicator of possible treatment non-adherence, although this relationship has not been directly validated in the current study. Since treatment non-adherence is associated with poor outcomes such as disease recurrence, treatment failure, and drug-resistant TB, monitoring submission patterns could help TB programs identify patients who may benefit from additional adherence support and timely interventions. This study seeks to address this knowledge gap by estimating the time to first and recurrent missed VDOT sessions and to identify factors associated with the timing of these missed sessions.

## Materials and methods

### Study design and setting

This was a retrospective cohort study utilizing secondary data from the DOT Selfie randomized controlled trial (RCT) conducted between July 2020 and October 2021. The parent trial was implemented across five TB clinics in Kampala, Uganda: Mulago National Referral Hospital, Lubaga Hospital, Kawaala Health Centre IV, Kitebi Health Centre IV, and Kisenyi Health Centre IV.

The DOT Selfie RCT was an open-label, two-arm randomized controlled trial that enrolled 144 adults aged 18-65 years with laboratory-confirmed, drug-susceptible TB, randomizing them 1:1 to VDOT or usual care DOT using permuted-block randomization stratified by sex. Participant recruitment took place between July 2020 and February 2021, with follow-up extending through October 2021. Each participant was followed for six months, and follow-up visits were conducted at months 2, 4, and 6. The current study included only participants from the VDOT arm, forming a single-cohort analysis.

### Data collection procedures and tools

At baseline, trained research assistants administered a structured questionnaire as described in the published protocol (9). The tool, informed by prior literature, was translated into Luganda and back-translated into English, thereby minimizing information bias. It captured information on TB diagnosis, sociodemographic characteristics, phone ownership and digital experience, transport challenges, social support, privacy concerns, and knowledge and perceptions of TB.

### VDOT procedures

Participants in the VDOT arm received a smartphone with a preinstalled HIPAA-compliant VDOT application and were trained on how to record and submit daily medication videos. The VDOT system consisted of three components: (1) a participant-facing smartphone application used to record medication intake videos, (2) a secure cloud-based server for encrypted storage of uploaded videos, and (3) a provider-facing web-based platform used by study staff to review submitted videos.

At enrollment, participants received face-to-face training on TB medication adherence and the use of the VDOT application. Participants were instructed to record themselves swallowing their daily TB medications and submit the videos through the application. Each submitted video was automatically encrypted, time-stamped, and uploaded to the secure cloud server, after which the video was automatically removed from the participant's smartphone. Automated daily SMS reminders were sent to participants to encourage medication intake and video submission.

On the provider side, trained study staff at the clinic logged into the secure VDOT system daily via a computer or tablet to download and review patients’ submitted videos, document medication adherence, and record any self-reported side effects. The protocol required staff to review videos within 24 hours of submission. Following review, study nurses were responsible for initiating follow-up contact, via phone call, within 24 hours of any missed video submission. If no response was received within 72 hours, the protocol escalated the follow-up to a field visit. Participants who remained unreachable after two weeks of active follow-up were expected to return during scheduled clinic visits, and persistent nonattendance was communicated to the national TB program staff for additional support. These procedures were intended to reinforce adherence and maintain patient engagement throughout treatment. Observer performance was monitored through the VDOT system's timestamped dashboard logs, which recorded when each video was accessed and reviewed.

### Study variables

The outcome variables were the time to first and recurrent missed VDOT session(s). For the first missed session, this was defined as the number of days from treatment initiation to the first missed video submission. For recurrent missed sessions, the outcome was the gap time defined as the number of days between consecutive missed videos.

Independent variables included background characteristics such as age, sex, education level, marital status, estimated monthly income, smoking status, alcohol use, HIV status, and digital literacy.

Digital literacy was assessed using a 14-item questionnaire evaluating the frequency and ease of using digital tools like smartphones and basic phone features. The items covered internet usage, social media, WhatsApp, phone calls, text messaging, photography, and video-taking. Responses were scored based on frequency (e.g., daily to never) and ease (e.g., very easy to very difficult), with a maximum possible score of 47. Participants were classified into low (<60%, score <28·2), moderate (60-84%, scores between 28·2 and 39·5), and high (≥85%, score ≥39·5) digital literacy categories. The scale demonstrated excellent internal consistency, with a Cronbach's alpha of 0·94, indicating reliable measurement.

### Sample size

The parent trial was powered on its primary outcome (the fraction of expected doses observed), and its full sample-size calculation is reported in the published protocol (9). The present study analyzed the complete VDOT arm; the unit of analysis is the individual participant (*n* = 71), and repeated missed-video events contributed by the same participant were handled using models with within-participant clustering, as described below.

Although the parent trial was not powered *a priori* for the specific factors examined here, the statistical power of the time-to-event analyses is governed by the number of events rather than the number of participants. A widely used rule of thumb for time-to-event models recommends observing at least ten outcome events for each covariate estimated, because it is the number of events, rather than the number of participants, that determines how much information the model has and how precisely each effect can be estimated (10). With no more than five covariates in any final model, this rule would require roughly 50 events; our analyses were based on well above this threshold, with 62 first missed sessions in the time-to-first analysis and 935 missed sessions in the recurrent-event analysis accrued over 12,922 expected submissions. We therefore interpret the descriptive time-to-event estimates as adequately supported, while the multivariable factors associated are presented as hypothesis-generating and are discussed with attention to the precision limits imposed by small exposure subgroups.

### Kaplan–Meier survival analysis

Data management and statistical analyses were conducted using Stata version 14·2 (Stata Corp LLC, College Station, TX, USA). To estimate the median time to the first missed VDOT session, the traditional Kaplan–Meier survival analysis was used, treating the first missed video as a terminal event. For estimating the median time to multiple missed videos (i.e., recurrent missed sessions), the conditional risk set model was applied. This approach accounts for repeated events (multiple missed videos by the same patient) and the time between consecutive missed sessions. In this model, the “clock” is reset after each failure (missed video), and time is measured from the previous missed session rather than continuously from the start of the study. This method captures the median time between missed sessions while accounting for within-patient correlations. The median times to both the first and multiple missed videos, along with quartiles. To assess whether the median time to missed videos differed across sociodemographic and clinical factors, the log-rank test was used to compare survival distributions.

### Cox proportional hazard model and the Prentice-Williams-Peterson Gap time model

To identify associated factors of the time to the first missed VDOT session, we used the traditional Cox Proportional Hazards (PH) model. Time to the first missed session served as the dependent variable. To identify factors associated with time to subsequent missed VDOT sessions, we used the Prentice-Williams-Peterson Gap Time (PWP-GT) model, which is an extension of the traditional Cox Proportional Hazards model. This model stratifies by event order and models the time between successive missed sessions, ensuring that a participant must experience event *k* before being at risk for event *k* *+* *1*. To account for within-participant correlation across recurrent events, robust standard errors clustered by participant were used in the PWP-GT model, implemented using the “cluster (patient_id)” option in Stata 14·2.

For both models, patients who remained adherent to VDOT, died, were lost to follow-up, or withdrew from the study were treated as censored observations. An initial bivariate analysis was conducted to explore associations between the outcome and the independent variables. Variables with a p-value ≤ 0·25 in the bivariate analysis, as well as those with established theoretical relevance, were considered for inclusion in the multivariable model. Multicollinearity among the selected variables was assessed using the Variance Inflation Factor (VIF), with a VIF > 5 considered indicative of significant multicollinearity. For multivariable analysis, we used a manual backward elimination procedure rather than relying on automated stepwise algorithms. This approach allowed for epidemiological reasoning at each step, guided by both statistical significance (Wald's test) and the theoretical importance of variables.

To assess confounding, we monitored changes in hazard ratios after the removal of each variable and adopted a conservative threshold of greater than 10% change to indicate potential confounding. None of the excluded variables produced such changes, suggesting that confounding was unlikely. Model fit was assessed using Cox-Snell residuals, under the expectation that the cumulative hazard function would follow an exponential distribution with a hazard rate of one if the model was well-fitted. Final results were reported as hazard ratios with 95% confidence intervals, and statistical significance was set at *p* < 0·05.

### Proportional hazard assumption

The proportional hazards assumption was tested using the global Schoenfeld residuals test and time-varying interactions for the key variables in the models. For Model 1 (single event) and Model 2 (recurrent event), the global test yielded p-values of 0·7327 and 0·5043, respectively, both greater than the conventional threshold of 0·05, indicating no violation of the proportional hazards’ assumption. Furthermore, the time-varying interactions for digital literacy, alcohol use, marital status, age category, and household size all had p-values greater than 0·05, suggesting that the effects of these variables do not significantly change over time. Therefore, we conclude that the proportional hazards assumption holds across both models (See Supplementary Table S1 in the Supplementary Materials).

## Results

### Participant follow-up

A total of 72 participants with drug-susceptible tuberculosis were initially enrolled in the VDOT arm of the DOT Selfie RCT. Of these, one participant did not initiate treatment and was excluded from follow-up analyses. The remaining 71 participants were followed prospectively during the study period. Among them, one participant died, two withdrew, one was lost to follow-up, and two remained on treatment at the end of the study period. Of the remaining 65 participants, three did not miss any video submissions during follow-up, while the other 62 missed at least one session. In total, nine participants were considered censored in the survival analysis of missed VDOT sessions. Of the 12,922 expected video submissions, 11,987 (92·8%) were successfully submitted and reviewed by a trained study nurse within 24 hours. A total of 935 missed video submissions were included in the recurrent event analysis. Participants missed an average of 14 VDOT sessions (SD = 16), with a median of 7 missed sessions (IQR: 2–22) and a range of 0 to 79 missed sessions per participant.

### Baseline characteristics of people with Tb who used vdot

Among the 71 participants, 36 (51%) were female. Twenty participants (28%) had previously been treated for TB, while 51 (72%) had no previous TB treatment. Twenty-three participants (32%) were living with HIV. Half (51%) of the participants had never been married. The median household size was 4 (IQR: 2–5), and the median estimated monthly income was USD 43 (IQR: 14–116) (Table 1).

**Table 1:** Summary of patient characteristics.

Variables	Summary measure, *N* = 71
Sex, n (%)	
Male	35 (49)
Female	36 (51)
Age, Median (IQR)	29 (24, 42)
Age category, n (%)	
≤30 years	40 (56)
>30 years	31 (44)
Highest level of education, n (%)	
No formal education or primary	27 (38)
Secondary	28 (39)
Tertiary	16 (23)
Marital status, n (%)	
Never married	36 (51)
Currently Married	22 (31)
Previously Married	13 (18)
Employment status, n (%)	
Employed	42 (59)
Unemployed	29 (41)
Household size, Median (IQR)	4 (2, 5)
Estimated monthly Income, Median (IQR)^a^	USD 43 (14, 116)
Reported alcohol use, n (%)	
No	60 (85)
Yes	11 (15)
Cigarette smoking status, n (%)	
Never smoked	60 (85)
Previously smoked or currently smoking	11 (15)
HIV status, n (%)	
Positive	23 (32)
Negative	48 (68)
Digital literacy, n (%)	
Low (<60%)	40 (56)
Moderate (60-84%)	20 (28)
High (≥85%)	11 (16)
TB treatment history, n (%)	
Previous TB treatment	20 (28)
No previous TB treatment	51 (72)

a 1 USD = 3,451 UGX.

### Median time to first missed VDOT session

**Figure 1:**
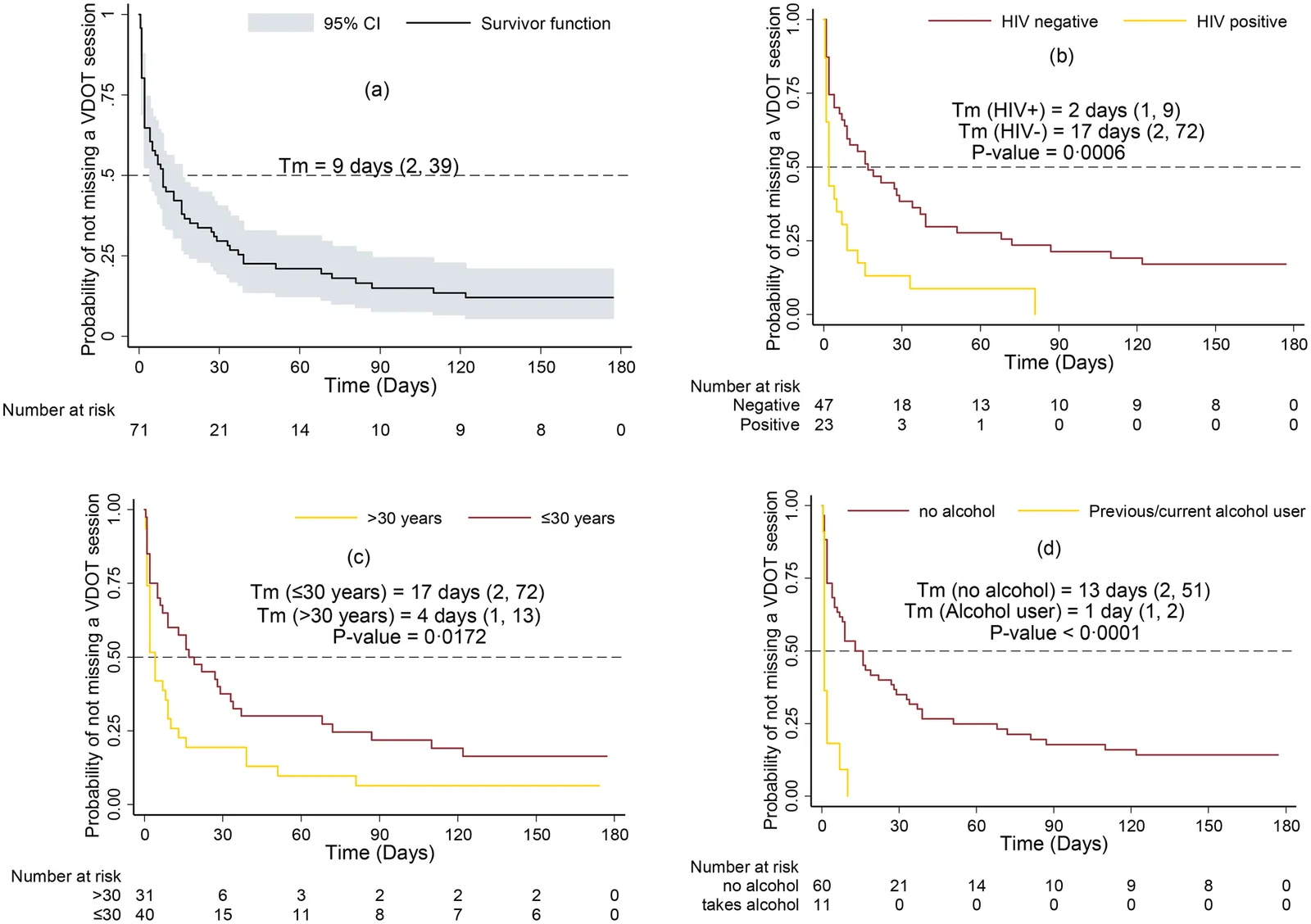
Kaplan–Meier survival curves showing median time to first missed VDOT session. **(a)** Overall survival, stratified by **(b)** HIV status, **(c)** Age category, and **(d)** Alcohol use. Tm means median time. The horizontal dashed reference line represents a 50% survival probability on the KM curve.

The overall median time to the first missed session was 9 days (2, 39). Participants co-infected with HIV missed VDOT significantly earlier than those without HIV (2 vs 17 days, p-value = 0·0006). Patients ≤30 years had a significantly longer time to first missed session (17 days, I2, 72) than those >30 years (4 days, 1, 13; p-value = 0·0172). Alcohol use was strongly associated with earlier missed sessions (1 day, 1, 2) compared to those not reporting alcohol use (13 days, 2, 51, p-value < 0·0001) (Figure 1).

### Median time to multiple missed VDOT sessions

The overall median time to the next missed session was 2 days (1, 8). Males missed another session sooner (1 day, 1, 7) than females (2 days, 1, 9, p-value = 0·0037). Participants living with HIV had shorter intervals between missed sessions than those without HIV (1 day, IQR: 1–6 vs. 2 days, IQR: 1–11; *p* < 0·0001). Patients on TB re-treatment missed sooner than those who were new to treatment (1 day, IQR: 1–5 vs. 2 days, IQR: 1–9; *p* = 0·0006). Alcohol use also predicted earlier recurrence (1 day, IQR: 1–4 vs. 2 days, IQR: 1–10 among non-users; *p* < 0·0001). Finally, younger participants (≤30 years) had longer intervals between missed sessions than with older participants (>30 years) (3 days, IQR: 1–10 vs. 1 day, IQR: 1–7; *p* = 0·0008) (See Supplementary Figure S1 in the Supplementary Materials).

### Factors associated with time to first missed VDOT session

Multivariable analysis showed people with TB living with HIV were 99% more likely to miss a VDOT session earlier during treatment compared to their HIV-negative counterparts (aHR = 1·99; 95% CI: 1·09, 3·62). Those who were currently married were 88% more likely to miss a session earlier than individuals who had never been married (aHR = 1·88; 95% CI: 1·03, 3·42). Participants who reported alcohol use were four times more likely to miss a session earlier than those who did not (aHR = 4·11, 95% CI: 1·89, 8·97) (Table 2).

**Table 2:** Simple and multivariable analysis for time to first missed VDOT session.

Variables	IR	cHR (95% CI) P-value	aHR (95% CI) P-value
Age category			
≤30 years	1·79	1·00	1·00
>30 years	4·38	1·79 (1·08, 2·97) *	1.02 (0.55, 1.88)
HIV status			
Positive	9·42	2·38 (1·38, 4·09) **	1.99 (1·09, 3·62) *
Negative	1·76	1·00	1·00
Sex			
Female	2·32	1·00	··
Male	2·66	1·22 (0·74, 2·01)	··
Marital status			
Never married	1·52	1·00	1·00
Currently Married	4·20	1·87 (1·06, 3·32) *	1·88 (1·03, 3·42) *
Previously Married	7·81	2·35 (1·20, 4·61) *	1·73 (0·80, 3·73)
Estimated monthly Income			
<USD 43	2·31	1·00	··
≥USD 43	2·64	1·11 (0·67, 1·84)	··
Household size	··	0·96 (0·87, 1·06)	··
Reported alcohol use			
Yes	40·0	4·46 (2·15, 9·21) ***	4·11 (1·89, 8·97) ***
No	2·06	1·00	1·00
TB treatment history			
Previous TB treatment	1·92	1·00	··
No previous TB treatment	2·75	1·03 (0·58, 1·83)	··
Digital literacy			
Low (< 60%)	2·92	2·84 (1·01, 8·05) *	2·08 (0·70, 6·16)
Moderate (60-84%)	3·51	2·96 (1·01, 8·71) *	2·60 (0·85, 7·93)
High (≥ 85%)	0·63	1·00	1·00

*P-value ≤ 0·05, **P-value ≤ 0·01, ***P-value ≤ 0·001, IR-Incidence rate per 100 person-days, cHR- crude Hazard Ratio, aHR- adjusted Hazard Ratio, I USD = 3,451 UGX.

### Factors associated with time to multiple missed VDOT sessions

In multivariable analysis, participants who were currently married were 34% more likely to experience earlier multiple missed VDOT sessions compared to those who had never been married (aHR = 1·34; 95% CI: 1·03, 1·75). In contrast, each additional household member was associated with a 4% lower likelihood of missing sessions earlier (aHR = 0·96; 95% CI: 0·92, 0·99) (Table 3).

**Table 3:** Simple and multivariable analysis for gap time between missed VDOT sessions.

Variables	IR	cHR (95% CI) P-value	aHR (95% CI) P-value
Age category			
≤30 years	7·52	1·00	1·00
>30 years	11·22	1·20 (0·96, 1·51)	1·16 (0·89, 1·51)
HIV status			
Positive	13·64	1·17 (0·92, 1·48)	1·13 (0·88, 1·45)
Negative	7·07	1·00	1·00
Sex			
Female	7·40	1·00	··
Male	11·47	1·09 (0·86, 1·36)	··
Marital status			
Never married	7·74	1·00	1·00
Currently Married	10·90	1·26 (0·94, 1·69)	1·34 (1·03, 1·75) *
Previously Married	10·58	1·25 (0·98, 1·59)	1·19 (0·94, 1·49)
Estimated monthly Income			
<USD 43	7·94	1·00	··
≥USD 43	10·75	1·05 (0·85, 1·30)	··
Household size	··	0·97 (0·94 1·01)	0·96 (0·92, 0·99) *
Reported alcohol use			
No	7·79	1·00	··
Yes	16·71	1·17 (0·88, 1·55)	··
TB treatment history			
Previous TB treatment	12·75	1·17 (0·91, 1·51)	1·10 (0·83, 1·46)
No previous TB treatment	8·27	1·00	1·00
Digital literacy			
Low (<60%)	9·43	0·88 (0·44, 1·74)	··
Moderate (60-84%)	8·60	1·03 (0·52, 2·07)	··
High (≥85%)	16·43	1·00	··

*P-value ≤ 0·05, IR-Incidence rate per 100 person-days, cHR- crude Hazard Ratio, aHR- adjusted Hazard Ratio, I USD = 3,451 UGX.

## Discussion

To our knowledge, this is the first study to estimate when missed video sessions occur as a marker of disengagement with the VDOT system and the factors predicting these lapses. Previous VDOT studies have focused solely on aggregated non-adherence rates and reported the cumulative missed videos (6, 8, 11, 12). Therefore, we discuss our findings within the broader context of previous research on other digital adherence technologies (DATs), such as 99DOTS and the pillbox, which involve patient engagement with digital medication monitoring (13–15). We found that the median time to the first missed session was nine days, suggesting that a substantial proportion of patients experienced a lapse in medication video submission within the first two weeks of treatment. Furthermore, the median interval between subsequent missed sessions was notably short, only two days, indicating a tendency toward clustered or repeated missed video submissions once an initial lapse occurred. This highlights the importance of close monitoring of patient engagement and continuous support, even when using remote VDOT programs.

Previous studies using other DATs besides VDOT have consistently demonstrated a decline in user engagement over time as a proxy for non-adherence (13, 14). In Uganda, adherence to 99DOTS declined from approximately 73% in the first month to 44% by the sixth month of treatment (13). Similarly, in India, a long-running deployment within Mumbai's public-sector TB program, covering 250 health centers, reported a drop in 99DOTS engagement from 74% on day one to 60% by month three and 56% by month six (14). Another study in North India, using data from the national TB database, found that 67% of participants never called the 99DOTS system throughout the study period, indicating substantial disengagement (11). A recent scoping review further emphasized variability in DAT adherence, showing that only 29% of participants maintained perfect engagement, with weighted average adherence rates of 60% for 99DOTS, 81% for video-supported treatment, and 87% for electronic pillboxes (15). This early decline suggests that VDOT programs should actively monitor and support patients from the start of treatment, particularly during the first few weeks, rather than assuming sustained engagement over time.

Several patient-related factors were associated with earlier missed video sessions. Participants co-infected with TB and HIV missed their first VDOT session significantly earlier than those without HIV infection. This finding is consistent with results from a study in Ethiopia, where people co-infected with TB and HIV were more likely to have missing digital confirmations from a smart pillbox adherence intervention compared to HIV-negative participants (16). The high pill burden from the concurrent use of ART and anti-TB medications can lead to treatment fatigue and scheduling difficulties, thus disrupting adherence to digital health tools. Additionally, studies from South Africa and Kenya have shown that individuals co-infected with TB and HIV often exhibit lower motivation to adhere to treatment (17, 18). This reduced motivation, compounded by emotional strain, forgetfulness, and fear of disclosing HIV status to family members, can further undermine adherence and engagement with digital adherence technologies. Taken together, these mechanisms indicate that co-infected patients are a priority group for early, tailored adherence support, and that integrating VDOT follow-up with existing TB-HIV services could help address the pill burden, motivational, and disclosure barriers that drive their earlier disengagement.

Reported alcohol use was significantly associated with early missed video submissions. Reported alcohol use may be associated with missed video submissions through several plausible pathways, including disrupted routines and effects on attention and memory that could reduce consistent engagement with daily video recording; however, our binary measure of alcohol use cannot establish a causal relationship or quantify consumption. Findings from our published study indicate that people with TB who reported alcohol use had a higher rate of missed VDOT sessions compared to non-alcohol users (12). This observation aligns with evidence from a systematic review, which found that alcohol use was associated with non-adherence to mobile health applications for the prevention or management of non-communicable diseases (19). Practically, this suggests that a brief alcohol screen at VDOT initiation could help programs flag patients who may need additional reminders or structured routines to sustain daily video submission, while recognizing that the direction and size of this association require confirmation in larger studies with graded measures of consumption.

Married individuals were more likely to miss VDOT sessions earlier than those who were not married. This finding was surprising because it contradicts the traditional belief that being married provides additional social support from a partner, which may promote better adherence to the VDOT engagement. However, privacy concerns arising from shared phone use between partners may compromise VDOT engagement, particularly when video submissions risk unintended disclosure of TB status (8, 20). A larger household size was significantly protective of earlier recurrent missed VDOT submissions. This may reflect the role of social support within households, where members can provide reminders, accountability, and emotional reinforcement that facilitate adherence to digital treatment monitoring. This observation is consistent with studies from Uganda and Ethiopia, which have highlighted the protective influence of family and community networks in supporting TB treatment engagement (16, 21). Future studies should examine the joint mechanisms through which marital dynamics and broader household structures influence engagement with VDOT.

### Strengths and limitations

A key strength of this study is that it is the first to apply a time-to-event approach to examine when people with TB miss VDOT sessions. This approach provided important insights into the timing and patterns of missed submissions, demonstrating that some patients were more likely to miss sessions early in treatment. These findings may help TB programs identify patients who require additional adherence support or more flexible, patient-centered care strategies. However, the study had several limitations. First, missing a VDOT session did not necessarily indicate a missed medication dose. For example, our previously published findings showed that approximately 15% of missed videos occurred among patients who reported taking their medication but forgetting to record or submit the video (4). In addition, the current study did not directly assess the relationship between missed VDOT submissions and clinical treatment outcomes. Therefore, missed video submissions should be interpreted as potential indicators of adherence challenges rather than definitive evidence of medication non-adherence or poor clinical outcomes. Future studies should evaluate whether VDOT submission gaps predict unfavorable TB treatment outcomes.

Second, VDOT adherence depends not only on patient participation but also on consistent provider engagement. Observer fatigue, particularly in low-resource settings with high patient-to-staff ratios, may threaten the long-term sustainability of VDOT programs. Delayed or absent review of submitted videos could reduce patient motivation if participants perceive that their efforts are not being acknowledged. In this study, several protocol-level safeguards were implemented to mitigate this risk. Study staff were required to review submitted videos within 24 hours, initiate follow-up phone contact within 24 hours of a missed video submission, and escalate follow-up to field visits if participants remained unreachable after 72 hours. Nevertheless, formal measures of observer performance, such as adherence to review timelines or the frequency of provider feedback, were not pre-specified study outcomes and therefore could not be evaluated.

Third, this analysis was constrained by the fixed size of the VDOT arm (*n* = 71). While the number of recurrent events supported the time-to-event modeling, several exposure subgroups were small, notably participants living with HIV (*n* = 23) and those reporting alcohol use (*n* = 11), which warrants cautious interpretation and requires confirmation in larger cohorts. In addition, alcohol use and cigarette smoking were captured as binary baseline measures inherited from the parent trial's questionnaire and therefore do not distinguish between light and heavy use, which may better reflect the underlying exposure. This would be preferable in future studies. Consistent with these constraints, the multivariable results are best regarded as hypothesis-generating signals to guide larger confirmatory work rather than definitive effect estimates.

## Conclusion

This study highlights factors that may limit the effective use of VDOT in Uganda. The findings highlight the need for rapid follow-up after an initial missed VDOT session to prevent further lapses in video submission. Efforts should focus on strengthening integrated TB–HIV services through tailored adherence counseling for co-infected patients. In addition, VDOT should be adapted to address the unique challenges faced by married individuals and incorporate differentiated support strategies for people with TB who use alcohol. Importantly, the effectiveness of VDOT depends not only on patient participation in daily video submission but also on timely provider review, follow-up, and ongoing adherence support. Strengthening accountability and engagement on both the patient and provider sides may improve the sustainability and effectiveness of VDOT implementation.

## Data Availability

De-identified data are available upon request, subject to approval and data use agreements with the University of Georgia and Makerere University. Requests to access these datasets should be directed to jsekandi@uga.edu.
