# Supplementary material for: Time to first and recurrent missed video directly observed therapy sessions and associated factors among people with Tuberculosis in Kampala, Uganda
Source: Front Digit Health. 2026 Sep 1;8:1815509. doi: 10.3389/fdgth.2026.1815509 (PMC13574809; doi:10.3389/fdgth.2026.1815509)
Supplement: Supplementary file 1 [file Datasheet1.docx]

**Supplemental Material**

**Table SI: Proportional Hazard Assumption Test Results**

|  | **Result** | |
| --- | --- | --- |
|  | **Model 1 (Single event)** | **Model 2 (Recurrent event)** |
| Global test | Chi (2) = 4·40, df = 7, P = 0·7327 | Chi (2) = 5·31, df = 6, P = 0·5043 |
| **Time-varying interaction** |  |  |
| Digital literacy | P-value = 0·256 |  |
| Alcohol consumption | P-value = 0·710 |  |
| Age category | P-value = 0·240 | P-value = 0·834 |
| Marital status | P-value = 0·187 | P-value = 0·426 |
| Household size |  | P-value = 0·581 |

**Fig S1: Kaplan–Meier Survival Curves Showing Median Time to Multiple Missed VDOT Session**

**Legend**

a) Overall

Stratified by:

b) Sex
c) HIV status
d) TB treatment history
e) Alcohol consumption
f) Age category
